# Eligibility Criteria Perpetuate Disparities in Enrollment and Participation of Black Patients in Pancreatic Cancer Clinical Trials

**DOI:** 10.1200/JCO.21.02492

**Published:** 2022-03-22

**Authors:** Andrea N. Riner, Selamawit Girma, Vignesh Vudatha, Nitai Mukhopadhyay, Nevena Skoro, Tamas S. Gal, Devon C. Freudenberger, Kelly M. Herremans, Thomas J. George, Jose G. Trevino

**Affiliations:** ^1^ University of Florida College of Medicine, Department of Surgery, Gainesville, FL; ^2^ Virginia Commonwealth University, Massey Cancer Center, Richmond, VA; ^3^ Virginia Commonwealth University, Department of Surgery, Richmond, VA; ^4^ Virginia Commonwealth University, Department of Biostatistics, Richmond, VA; ^5^ University of Florida College of Medicine, Department of Medicine, Division of Hematology and Oncology, Gainesville, FL

## Abstract

Clinical trials determine safety and efficacy of cancer therapeutics and establish standards of care. Minority patient participation in cancer clinical trials is dismal. We aimed to determine the impact of eligibility criteria on disparities in pancreatic ductal adenocarcinoma (PDAC) clinical trial candidacy.

**METHODS:**

Traditional PDAC trial eligibility criteria were obtained from ClinicalTrials.gov. Patients with PDAC who sought care at Virginia Commonwealth University Health from 2010 to 2019 were included. Clinical data were obtained from billing codes and discrete values in the electronic medical record. Eligibility criteria differences between racial groups were determined using chi-squared tests and unconditional maximum likelihood-based odds ratios.

**RESULTS:**

Among 676 patients, most identified as Black or White race (42.5% and 51.6%, respectively). Using traditional criteria, Black patients were more likely to be ineligible for participation compared with White patients (42.4% *v* 33.2%, *P* = .023) secondary to hypoalbuminemia (14.1% *v* 7.9%, *P* = .023), HIV (3.1% *v* 0.3%, *P* = .010), hepatitis B (1.7% *v* 0%, *P* = .043), and hepatitis C (9.1% *v* 3.4%, *P* = .005). Black patients were also numerically more likely to be ineligible because of renal dysfunction, recent coronary stenting, and uncontrolled diabetes mellitus. Prior cancer treatment excluded fewer Black than White patients (9.1% *v* 14.0%, *P* = .072), most attributable to lower rates of neoadjuvant chemotherapy received. Strategic eligibility criteria revisions could equalize ineligibility rates between Black and White patients (26.8% *v* 24.8%, *P* = .581).

**CONCLUSION:**

Traditional eligibility criteria differentially exclude Black patients from participating in PDAC clinical trials. These criteria perpetuate disparities, limit generalizability, and are often not medically justifiable. Revised criteria may improve participant diversity, without compromising safety or study results.

## INTRODUCTION

Clinical trials investigating novel treatments for pancreatic ductal adenocarcinoma (PDAC) are troubled with under-representation of diverse participants. After adjusting for disease prevalence, Black, Asian or Pacific Islander, American Indian or Alaskan Native, and Hispanic patients have been significantly under-enrolled in PDAC clinical trials in the United States.^1^ In 2017, Congress issued a federal regulation (42 CFR Part 11) that led to increased reporting of clinical trial participants' race/ethnicity.^2^ However, enrollment of more diverse participants has not been demonstrated.^1^ Thus, the standard of care in cancer treatment is informed by studies conducted with predominantly non-Hispanic White participants.

**CONTEXT**

**Key Objective**
To determine the impact of eligibility criteria on disparities in pancreatic cancer clinical trial candidacy among Black and White patients.
**Knowledge Generated**
In this cross-sectional study, common eligibility criteria for phase II and III trials were applied to a cohort of patients with pancreatic cancer from a single institution to simulate the clinical trial screening process, demonstrating that Black patients had an overall rate of eligibility that was 9% lower than White patients, mostly attributable to malnutrition and infectious diseases. By implementing medically reasonable revised criteria, rates of ineligibility decreased for both Black and White patients, but notably equalized ineligibility rates between racial groups, thereby eliminating the eligibility gap on the basis of medical conditions and organ dysfunction.
**Relevance**
Revision of eligibility criteria is one potential means to improve representation of diverse patients in clinical trials and advance the generalizability of results to reflect real-world treatment of pancreatic cancer.

In addition to social justice concerns about equitable access to investigational therapeutics, there are biologic reasons necessitating diverse participation in trials. Drug-metabolizing enzymes have different distributions in African, Asian, and White populations, influencing pharmacokinetics.^3-5^ Somatic and germline mutations, as well as alternative RNA splicing, vary among populations, which may affect pharmacokinetics, therapeutic resistance, response, and toxicity to targeted therapies.^6-8^ Postmarketing data revealing divergent efficacy and toxicity profiles have even led to race-specific/ethnicity-specific dosing guidelines.^6,7^ Although currently actionable molecular alterations are uncommon,^9^ matching targeted therapies to actionable alterations through precision medicine can improve survival.^10^ However, knowledge of possible differences in molecular profiles across populations is limited by inclusion of racial/ethnic minority participants in studies. Lack of diverse trial participants leaves providers with incomplete data on safety and efficacy of cancer therapeutics, potentially exacerbating disparities in survivorship, where one-year relative survival in 2017 was lower for Black patients (58.1% localized, 51.7% regional, and 15.3% distant) compared with White patients (63.9% localized, 58.5% regional, and 21.8% distant), regardless of stage.^11^

Although the reasons for under-representation in clinical trials are complex (mistrust in the medical system, systemic racism, differential access to care and centers conducting clinical trials, socioeconomic factors, lack of diversity among clinicians conducting trials, implicit bias, etc), one factor that has not been fully investigated is eligibility criteria.^12-14^ Intended to standardize participation for efficacy assessments and minimize risks, these criteria are set by trial sponsors but supported by regulatory authorities. However, some medical conditions may preferentially exclude minorities from trial participation without strong medical rationale. ASCO and Friends of Cancer Research (Friends), in collaboration with the US Food and Drug Administration (FDA), issued statements in 2017 to address the concern that restrictive eligibility criteria lead to reduced generalizability of study results. Modernized criteria were proposed, with revised guidelines on organ dysfunction, prior or current malignancy, and comorbidities.^15-17^ These statements highlighted the importance of expanded eligibility criteria for experimental therapies, but fell short of acknowledging the effects of restrictive criteria on racial/ethnic disparities in trial participation. To our knowledge, the magnitude of potential impact of expanded eligibility criteria on improving racial/ethnic disparities in eligibility has not been well studied. We sought to investigate the impact of traditional criteria on potential trial participation by race/ethnicity among a diverse patient population with PDAC. We hypothesize that traditional eligibility criteria lead to racial/ethnic disparities in potential participation in PDAC clinical trials and that eligibility among racial/ethnic minority patients may be significantly improved by implementing selectively less restrictive criteria.

## METHODS

This cross-sectional study was conducted at Virginia Commonwealth University (VCU) National Cancer Institute (NCI)–designated Massey Cancer Center and was performed in accordance with the Strengthening the Reporting of Observational Studies in Epidemiology (STROBE) reporting guidelines^18^ with VCU Institutional Review Board approval (IRB HM20022715).

### Selection of Patients

The Massey Cancer Center Registry was queried to retrospectively identify patients age ≥ 18 years diagnosed with PDAC on the basis of International Classification of Disease-Oncology histology codes and who received care at VCU between January 1, 2010, and December 31, 2019.

### Eligibility Criteria

**FIG A1.**
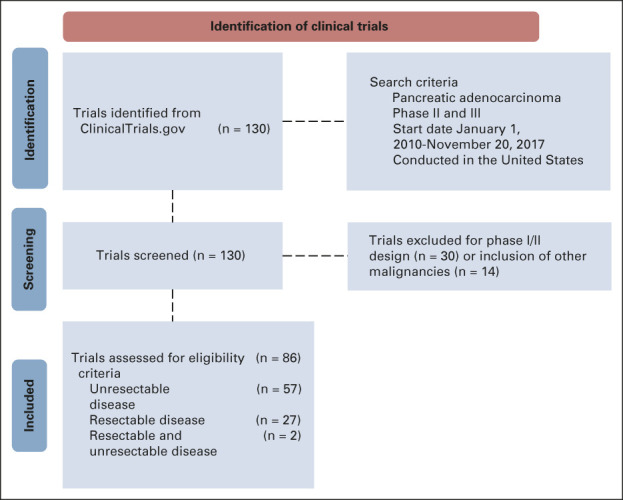
Pancreatic cancer clinical trials Preferred Reporting Items for Systematic Reviews and Meta-Analyses (PRISMA) diagram. Trials registered in ClinicalTrials.gov were reviewed to identify commonly used eligibility criteria to screen patients for possible enrollment.

We searched ClinicalTrials.gov for US phase II and III clinical trials enrolling patients with pancreatic adenocarcinoma, from January 1, 2010, through November 20, 2017 (ASCO-Friends' updated guideline publication date). Trials were selected (Appendix Fig A1, online only), and their eligibility criteria were obtained. Inclusion and exclusion criteria common across trials were noted. Criteria were assessed for objectivity, discrete values, or billing codes that would be readily available in the medical record, resulting in a final list of eligibility criteria (Appendix Table A1, online only) to be applied for a simulated patient screening process. Criteria were reviewed by a medical and a surgical oncologist to ensure that appropriate definitions were included, particularly when the criterion was not specifically defined. For example, we defined uncontrolled diabetes mellitus as HgbA1c ≥ 10% or median serum glucose ≥ 240 mg/dL within 90 days of the first oncology appointment. If multiple laboratory values were available, the healthiest (maximum) value was chosen for albumin, whereas the healthiest (minimum) value was chosen for creatinine. In the analysis of revised criteria, for patients with available creatinine results, but without creatinine clearance (CrCl), a higher threshold of 2.0 was implemented. CrCl was then calculated using the Cockcroft-Gault equation, and patients were reclassified as eligible if calculated CrCl was ≥ 30 mL/min.^19^ Autoimmune disorders were removed from the final analysis because of low case numbers (polyarteritis nodosa [n = 1], Hashimoto's thyroiditis [n = 1], autoimmune hepatitis [n = 1], scleroderma [n = 3], and systemic lupus erythematosus [n = 4]). Prolonged QTc was removed because of the high rate of missing data (n = 516, 81.1%). History of illicit substance use or alcohol abuse and uncontrolled psychiatric illness were removed because of subjectivity and risk for bias in reporting.

### Simulated Clinical Trial Screening and Statistical Analysis

The date of first encounter with clinical oncology was designated as the baseline date. The time frame for each criterion (Appendix Table A1) was determined in relation to this date. Data pertaining to exclusion criteria on the basis of serologic results and comorbid conditions were obtained from medical records and billing claims. Eligibility criteria were applied to each patient. Revised criteria (Table 1) were adapted from ASCO and Friends guidelines^15,16^ and FDA guidelines released in 2020^20^ and further refined on the basis of clinical judgment by physician members of the study team. Revised criteria were similarly applied to determine the racial distribution of eligible patients with less restrictive criteria. Percentage of ineligible patients were computed in each racial group, and unconditional maximum likelihood-based odds ratios (ORs) were calculated. Statistical significance of the difference in the proportion of ineligibility was assessed using Pearson's chi-squared test. Counts in each group were large enough that small sample size corrections were unnecessary. All analyses were performed using R v3.6.3 Statistical Software Package.^21^

**TABLE 1.**
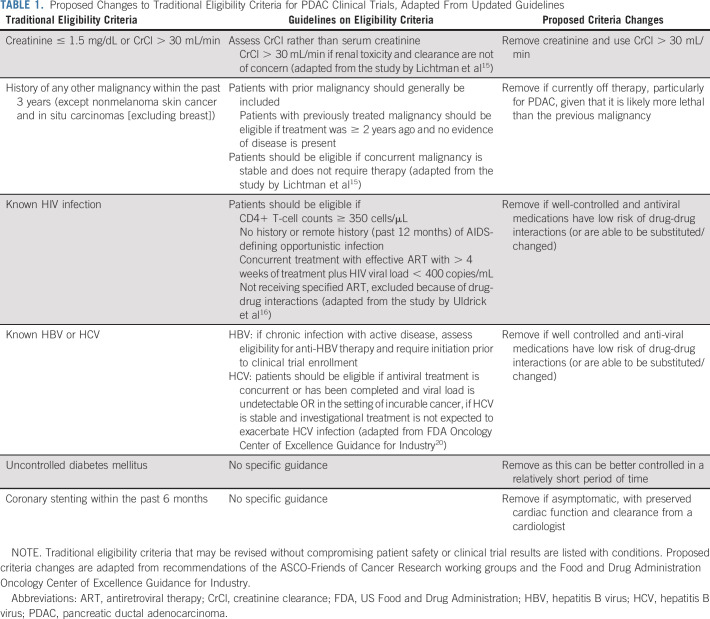
Proposed Changes to Traditional Eligibility Criteria for PDAC Clinical Trials, Adapted From Updated Guidelines

## RESULTS

### Patient Population

A total of 676 patients with PDAC were identified, with 287 Black (42.5%) and 349 White (51.6%) patients comprising the majority (n = 636; Table 2). All other racial/ethnic groups were relatively small and removed from further analysis. There was a slight male predominance (51.9%), and the average patient age was 65.9 (SD = 11.1) years. Approximately half (51.8%) were insured by Medicare. The majority (57.7%) had clinical stage III/IV disease. Compared with White patients, Black patients were significantly younger (64.2 *v* 67.2 years; *P* < .001) and more likely to be female (53.3% Black *v* 44.7% White, *P* = .037; Table 2). Insurance coverage of Black patients differed significantly from that of White patients (*P* = .012), with the greatest difference attributable to Medicaid-insured (10.5% Black *v* 4.0% White) and uninsured (10.1% Black *v* 5.4% White). The clinical disease stage distribution differed significantly, with 64.8% of Black patients having stage III/IV disease versus 51.5% of White patients (*P* = .003). Lack of a similar trend in pathologic stage may be attributed to 69.7% of data unknown or missing, reflective of metastatic disease where primary tumor was not resected or diagnosis of recurrent disease on the basis of imaging/clinical assessment.

**TABLE 2.**
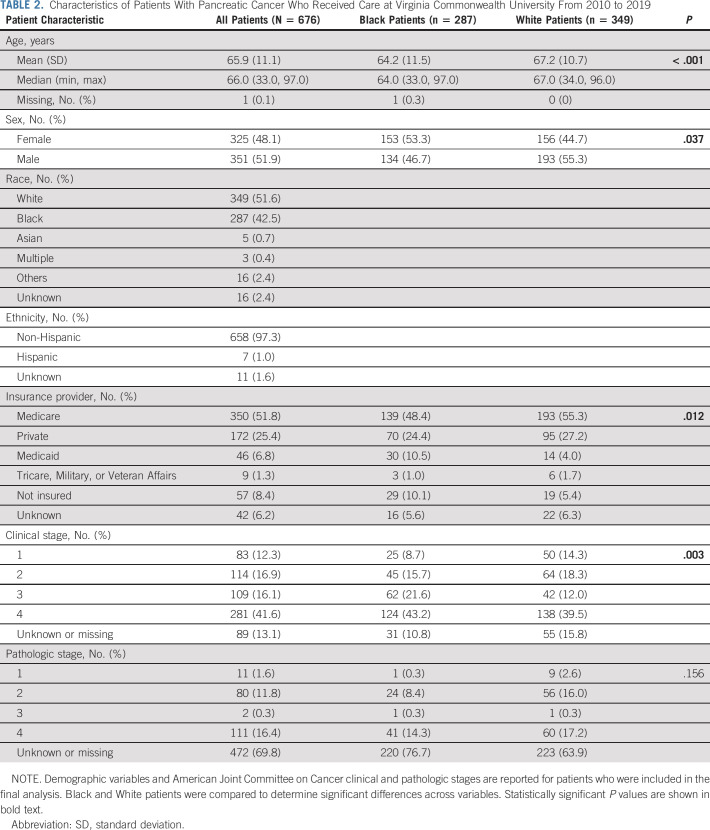
Characteristics of Patients With Pancreatic Cancer Who Received Care at Virginia Commonwealth University From 2010 to 2019

### Racial Differences in Eligibility With Traditional Criteria

**FIG 1.**
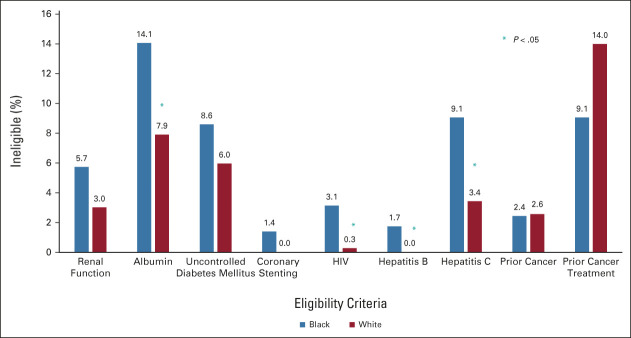
Clinical trial ineligibility for select traditional criteria, by racial group. The percentage of patients deemed ineligible for clinical trial participation on the basis of individual criterion are shown by race. Albumin, HIV, hepatitis B, and hepatitis C contributed significantly to higher rates of ineligibility for Black patients compared with White patients (*P < .05).

When traditional trial eligibility criteria were applied, Black patients were significantly more likely to be ineligible on the basis of hypoalbuminemia (OR, 1.90; 95% CI, 1.12 to 3.25), HIV (OR, 11.30; 95% CI, 1.42 to 89.7), or hepatitis C (OR, 2.81; 95% CI, 1.39 to 5.67) infections (Fig 1 and Table 3). Prior hepatitis B infection led to disqualification of 1.7% of Black patients, but no White patients were excluded (*P* = .043). Similarly, coronary stenting within the past 6 months excluded 1.4% of Black patients, whereas no White patients were excluded (*P* = .087). There was no difference in trial eligibility among Black and White patients because of renal dysfunction (OR, 1.95; 95% CI, 0.87 to 4.38) or uncontrolled diabetes mellitus (OR, 1.48; 95% CI, 0.80 to 2.74; Fig 1 and Table 3). Previous cancer rates were similar between Black (2.4%) and White (2.6%) patients; however, prior cancer treatment numerically excluded more White than Black patients (14.0% *v* 9.1%, *P* = .072; Fig 1 and Table 3). This was attributable to more White patients initiating neoadjuvant chemotherapy for PDAC before seeking care at VCU. No patients met exclusion criteria of body mass index > 55 kg/m^2^, concurrent pregnancy, or breastfeeding.

**TABLE 3.**
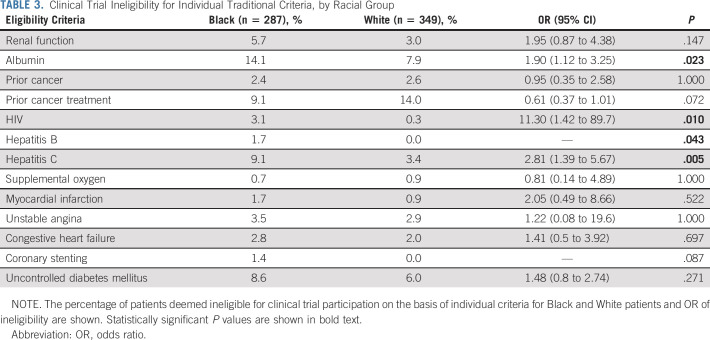
Clinical Trial Ineligibility for Individual Traditional Criteria, by Racial Group

### Impact of Alternative Revised Eligibility Criteria

**FIG 2.**
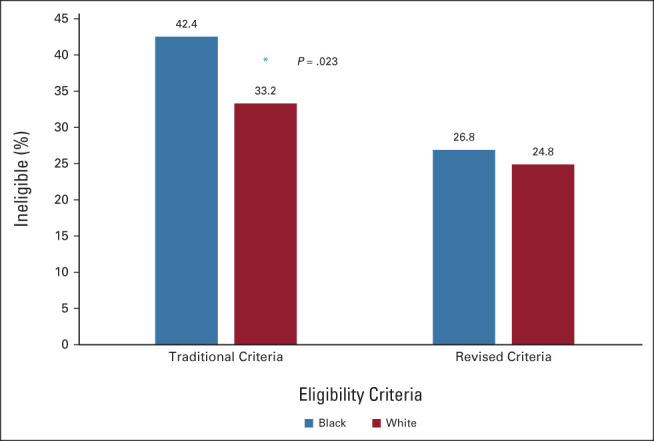
Overall clinical trial ineligibility for traditional and revised criteria, by racial group. Traditional criteria led to a significantly higher percentage of Black patients being ineligible, but revised criteria eliminated this disparity (*P = .023).

With traditional criteria, Black patients were more likely to be ineligible for participation compared with White patients (42.4% *v* 33.2%, *P* = .023; Fig 2). Revised criteria (Table 1) included removing historical, controllable, or manageable medical conditions including HIV, hepatitis C virus, hepatitis B virus, diabetes mellitus, previous cancer, and coronary stenting. After applying revised criteria, there was no longer any difference in ineligibility rates for Black or White patients (26.8% *v* 24.8%, *P* = .581; Fig 2).

## DISCUSSION

The results of this study demonstrate that traditional eligibility criteria for PDAC clinical trials disproportionately exclude Black patients from eligibility on the basis of medical conditions. Although these criteria are intended to reduce risk and define a homogenous study population, they have potential to create bias. Restrictive criteria not only limit generalizability of results to the healthiest patients, but many medical conditions that exclude patients from eligibility are also associated with health disparities. Infectious diseases, such as HIV and hepatitis, disproportionately affect Black patients.^22-25^ Similarly, chronic kidney disease, diabetes mellitus, heart disease, and obesity are more prevalent in Black and Hispanic populations.^26-31^ This leads to a double-hit phenomenon whereby patients with PDAC from minority backgrounds are less healthy and unlikely to be provided opportunities to participate in trials. Through revised eligibility criteria, there is potential to improve equitable eligibility for clinical trial participation, thus producing more generalizable results and reducing disparities in access to treatment.

To date, few studies have investigated the effect of eligibility criteria on enrollment of underserved populations. A study conducted at Howard University, in which consecutively diagnosed African-American patients with cancer were assessed for clinical trial eligibility, found that only 8.5% of patients were eligible, and among those ineligible, 17.1% were due to comorbidities.^32^ Our results likely overestimate eligibility as we applied only a subset of criteria discretely available from the medical record. In comparing ineligibility rates between Black and White patients, results have varied. A study conducted at an NCI-designated cancer center reported no difference in clinical trial eligibility on the basis of race/ethnicity.^33^ However, Langford et al^34^ investigated patients treated at 16 NCI Community Cancer Center Program sites from 2009 to 2012, finding that non-Hispanic Black patients were approximately 1.5 times more likely to be ineligible for clinical trial participation than non-Hispanic White patients because of comorbidities, but not abnormal labs or organ dysfunction. Penberthy et al^35^ evaluated reasons for ineligibility in cancer clinical trials from 2006 to 2010, reporting that comorbidities were the most common reason for ineligibility. Notably, they found no difference in rates of ineligibility between African American and White patients on the basis of comorbidities. However, African Americans were more likely to be ineligible because of mental status and anticipated noncompliance, highlighting the possible impact of implicit bias with subjective eligibility criteria.^35^ Although few studies assessing differential clinical trial eligibility for Black patients have identified discrepant contributing factors, racial/ethnic disparities in eligibility were consistent.

Differences in demographics, insurance provider, and disease stage between Black and White patients in our study were not surprising as Black patients tend to be diagnosed with PDAC at a younger age and more advanced stage.^36^ This aligns with fewer Black patients having Medicare coverage. Higher rates of Medicaid and no insurance coverage among Black patients likely reflect higher rates of financial vulnerability. Even if medically eligible, variable Medicaid coverage of testing and treatment within clinical trials may contribute to disparities in participation. In our study population, rates of renal dysfunction and diabetes mellitus were not significantly higher in Black compared with White patients. Higher prevalence of infectious diseases among Black patients in this study is consistent with national data,^22-25^ but in the era of highly effective therapies to control or cure these conditions, the absolute contraindication may be reconsidered. The condition independently leading to the highest number of ineligible Black patients was hypoalbuminemia, suggesting more malnutrition, poor protein nutritional intake, or renal-associated protein loss in Black patients. The rationale for ineligibility because of hypoalbuminemia is reasonable, but higher rates among patients with PDAC, particularly Black patients, underscore the need for nutritional optimization and effective therapies for cancer-associated cachexia.^37^ Given the lethality of PDAC, real-world treatment would not necessarily be withheld for medical conditions such as hypoalbuminemia, diabetes mellitus, HIV, hepatitis, or some cardiovascular diseases. Indeed, newer FDA-approved therapies for PDAC are administered to patients despite the absence of such patients in registration enabling clinical trials.^38^ Rather, specialists who can manage comorbidities may allow for safe enrollment of these patients. These results are likely translatable to clinical trials for other cancer types as they reflect common eligibility criteria barriers that are not specific to PDAC.

The exclusion criterion of uncontrolled diabetes mellitus is particularly troubling in PDAC trials. Some pancreatic tumors are diabetogenic, and surgical resection through pancreatectomy can contribute to endocrine insufficiency, which may be challenging to tightly control. We propose that diabetes mellitus status should not exclude any patient from a clinical trial if they are agreeable to close glucose management by a nononcology specialist or primary care provider, concurrent with anticancer therapy. Diabetes mellitus can be well controlled in a relatively short period of time for a majority of patients. However, poorly controlled diabetes mellitus may reflect the impact of underlying social determinants of health and inadequate access to care, and thus, clinical trial centers may need to facilitate diabetes mellitus care with providers in local communities or via telehealth. Regarding recent coronary stenting, if the patient is asymptomatic with adequate cardiac function, it is medically reasonable to allow for participation with cardiology approval. Infectious diseases, such as HIV and hepatitis, are successfully managed and suppressed with antiviral medications, and patients are now experiencing near-normal life expectancy. Collaboration with infectious disease experts is warranted to determine patient eligibility, with consideration of their disease status and alternative antiviral medications with less drug-drug interactions. Regarding renal dysfunction, controversy surrounds the use of historical markers of renal function that contain race modifiers or were developed from cohorts lacking diverse patients.^39^ Black patients tend to have higher baseline serum creatinine, possibly attributable to differences in glomerular filtration rates (GFR), tubular secretion of creatinine, and body composition affecting endogenous creatinine generation.^40-42^ Creatinine clearance, an estimate of GFR, has been considered the standard measure of renal function;^43^ however, these calculations are imperfect and updated measures are being standardized.^39,44^ Regardless, if a study therapy is not affected by renal metabolism or excretion, reasonable renal function should support trial participation. The summative effect of removing or modifying these criteria is profound and has potential to reduce bias in offering more equitable patient participation. Other barriers to participation include adequate insurance coverage, which may necessitate policy reform, and access to clinical trials. Hybrid or decentralized study designs, bringing trials to the patients, may improve accessibility.^45,46^ Although community engagement to increase awareness and acceptance of clinical trials continues,^47^ if eligibility criteria disproportionately exclude patients on the basis of comorbidities, then the impact on achieving equitable representation will continue to fall short of our goals.

Eligibility criteria standardization is shared by multiple stakeholders, extending beyond individual researchers' influence. Updated statements acknowledging the impact of eligibility criteria on racial/ethnic disparities are justified. Patient advocacy groups and professional societies have the voice to demand policy change. Funders should require eligibility criteria justification before providing support. Regulatory authorities should scrutinize the medical necessity of eligibility criteria. Stakeholders should be held accountable for their role as collective effort can create impactful results.

This study is subject to information bias given its retrospective design. Objective definitions were created for criteria if not stated explicitly on ClinicalTrials.gov, but these definitions may not accurately reflect the real-world interpretation by other clinicians. Data were limited to billing codes and discrete values within the medical record, contributing to possible information bias and subsequently misclassification of eligibility. Subjective eligibility criteria including illicit substance use, alcohol abuse, and uncontrolled psychiatric illness were removed from this analysis, which likely underestimates the impact that implicit bias adds to enrollment bias. As a single-center study, our findings may not be representative of other settings. However, the catchment area of VCU is richly diverse, with Black patients comprising a large proportion of the population. Our analysis of Hispanic/Latinx patients and other minority racial groups was limited by few cases and was not the focus of this analysis. The list of eligibility criteria selected for this simulated study was not comprehensive and may overestimate true eligibility in the real-world setting although our results likely reflect the majority of conditions that may contribute to disparities in eligibility. Similarly, the revised criteria proposed will need to take into consideration the unique toxicities and risks associated with novel therapeutics and may not represent a one-size-fits-all approach to disparity reduction. Finally, this work establishes the foundation that eligibility criteria may contribute to racial disparities in clinical trial participation, paving the way for future investigation on trends in modernized eligibility criteria implementation and their influence on disparities in eligibility, which extends beyond the scope of this study.

In conclusion, traditional clinical trial eligibility criteria disproportionately exclude Black patients, leading to reduced opportunities to participate in PDAC clinical trials. These restrictive criteria perpetuate disparities in clinical trial participation, limit the generalizability of results, and may not be medically justifiable. Careful consideration of the medical necessity of each criterion is needed on a trial-by-trial basis. In addition, more input from medical specialists may be indicated for the assessment of benefit versus risk for patient participation and comanagement throughout the trial. Together, these could have a profound effect on increasing eligibility of underserved populations, reducing disparities in clinical trial participation, and creating results that are more reflective of the patients that we serve.

## Figures and Tables

**TABLE A1.**
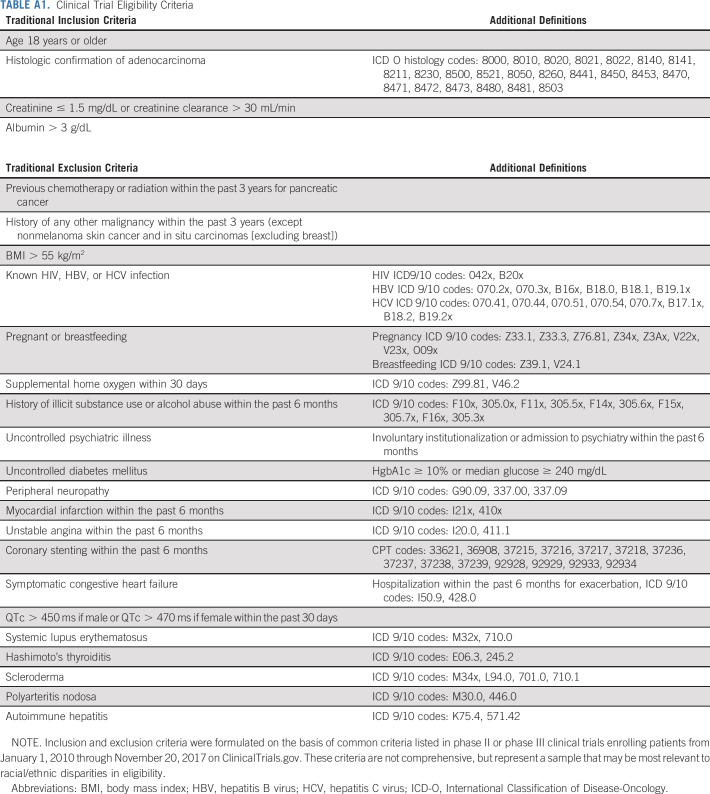
Clinical Trial Eligibility Criteria
